# P15 - Atopy and acute urticaria in childhood: is there an association?

**DOI:** 10.1186/2045-7022-4-S1-P70

**Published:** 2014-02-28

**Authors:** George N Konstantinou, Stefania Totska, Dimitra Georgiadou, Maria Farini, Alexandra Terzi, Despina Tsonoglou, Rozalia Valeri

**Affiliations:** 1 424 General Military Training Hospital, Thessaloniki, Greece; 2 First Pediatric Department, Aristotle University of Thessaloniki, Hippokration General Hospital, Thessaloniki, Greece

## Background and objective

Acute urticaria (AU) is a common condition that often presents in childhood. In this questionnaire-based prospective study we aim to investigate if there is any association between AU and atopy.

## Methods

Children up to 14 years of age presented with AU in the emergency department (ED) of a reference children’s hospital in the area of Thessaloniki were prospectively recorded. ED pediatricians filled out a relevant questionnaire per patient in the context of the taken personal medical history. Records included any doctor-diagnosed allergic disease and information derived from questions based on the International Study of Asthma and Allergies in Childhood (ISAAC) to identify any current or past allergic disease. Demographics and AU characteristics were additionally recorded.

## Results

256 AU cases (138 boys), 5.1±3.3 years old were recorded. Prevalence of AU did not differ with regards gender (*P*=0.376). The most common potentially associated triggers were food consumption, infections, hymenoptera stings, and vaccination. Atopy was not found to influence AU prevalence (*P*=0.156) or co-occurrence of angiooedema (*P*=0.226) which presented in 35.7% of the AU patients. Moreover angiooedema occurred independently of gender (*P*=0.085), with a trend to be more prominent in older children ages (*P*=0.061) and frequently associated with hymenoptera stings (*P*=0.001) but not be so frequent in the presence of an infection (*P*=0.005).

## Conclusions

Atopy does not appear to predispose to acute urticaria with or without angiooedema in children.

